# Regulation of molecular clock oscillations and phagocytic activity via muscarinic Ca^2+^ signaling in human retinal pigment epithelial cells

**DOI:** 10.1038/srep44175

**Published:** 2017-03-09

**Authors:** Rina Ikarashi, Honami Akechi, Yuzuki Kanda, Alsawaf Ahmad, Kouhei Takeuchi, Eri Morioka, Takashi Sugiyama, Takashi Ebisawa, Masaaki Ikeda, Masayuki Ikeda

**Affiliations:** 1 Graduate School of Science and Engineering, University of Toyama, 3190 Gofuku, Toyama city, Toyama 930-8555, Japan; 2 Graduate School of Innovative Life Science, University of Toyama, 3190 Gofuku, Toyama city, Toyama 930-8555, Japan; 3 Advanced Core Technology Department, Research and Development Division, Olympus Co. Ltd., 2-3 Kuboyama, Hachioji, Tokyo 192-8512, Japan; 4 Department of Psychiatry, Tokyo Metropolitan Police Hospital, 4-22-1 Nakano, Nakano-ku, Tokyo 164-8541, Japan; 5 Department of Physiology, Saitama Medical University, 38 Morohongo, Moroyama, Iruma-gun, Saitama 350-0495, Japan; 6 Molecular Clock Project, Project Research Division, Research Center for Genomic Medicine, Saitama Medical University, 1397-1 Yamane, Hidaka city, Saitama, 350-1241, Japan

## Abstract

Vertebrate eyes are known to contain circadian clocks, however, the intracellular mechanisms regulating the retinal clockwork remain largely unknown. To address this, we generated a cell line (hRPE-YC) from human retinal pigmental epithelium, which stably co-expressed reporters for molecular clock oscillations (*Bmal1-luciferase*) and intracellular Ca^2+^ concentrations (*YC3.6*). The hRPE-YC cells demonstrated circadian rhythms in *Bmal1* transcription. Also, these cells represented circadian rhythms in Ca^2+^-spiking frequencies, which were canceled by dominant-negative *Bmal1* transfections. The muscarinic agonist carbachol, but not photic stimulation, phase-shifted *Bmal1* transcriptional rhythms with a type-1 phase response curve. This is consistent with significant *M3* muscarinic receptor expression and little photo-sensor (*Cry2* and *Opn4*) expression in these cells. Moreover, forskolin phase-shifted *Bmal1* transcriptional rhythm with a type-0 phase response curve, in accordance with long-lasting CREB phosphorylation levels after forskolin exposure. Interestingly, the hRPE-YC cells demonstrated apparent circadian rhythms in phagocytic activities, which were abolished by carbachol or dominant-negative *Bmal1* transfection. Because phagocytosis in RPE cells determines photoreceptor disc shedding, molecular clock oscillations and cytosolic Ca^2+^ signaling may be the driving forces for disc-shedding rhythms known in various vertebrates. In conclusion, the present study provides a cellular model to understand molecular and intracellular signaling mechanisms underlying human retinal circadian clocks.

Daily behavioral and physiological rhythms are governed by the circadian clock system, which is composed of multiple oscillators in the body. The master circadian clock is located in the hypothalamic suprachiasmatic nucleus (SCN) in mammals^1^,^2^, which organizes rest of oscillators and ultimately coordinates the system’s circadian rhythms^3^. In addition, as in the lower vertebrate clock^4^, the mammalian eye contains a complete circadian clock^5^. For example, photoreceptor disc shedding^6^,^7^,^8^, dopamine synthesis^9^, and retinal electrical responses to light^10^ are all under the control of the circadian clock. Notably, melatonin release from cultured retina represented temperature-compensated circadian rhythms and could entrain to the light-dark cycles^11^,^12^,^13^, proving that the mammalian retina contains a self-sustaining and functional circadian clock. Consistently, clock gene expressions have been identified in the inner layer of the mammalian retina^14^,^15^,^16^, retinal ganglion cells^17^, Müller cells^18^ and retinal pigmental epithelium (RPE) cells^19^,^20^. Within various peripheral (i.e., non-SCN) circadian clocks, the importance of the clock in the eye should be emphasized as its exceptional role for the photic input (i.e., resetting) system to the central SCN clock.

Microarray assays have demonstrated that nearly 300 genes display circadian transcriptional activities within the eye^21^. Of the many molecular oscillators, the clock gene *Bmal1* may play a pivotal role in the retina, because a conditional knockout of *Bmal1* in the retina using CHX10-Cre resulted in a loss of circadian rhythm of inner retinal electrical activity in response to light^21^. Conversely, CHX10-Cre might not knockout *Bmal1* in RPE cells, because CHX10 is a transcriptional factor localized to the inner nuclear layer, particularly in bipolar cells^22^,^23^. Thus, it is still unknown how clock gene oscillations in RPE cells^19^,^20^ contribute to physiological rhythm generations in the eye. Because disc shedding of photoreceptor outer segments (OS) is mediated largely by phagocytic activities of RPE cells^24^,^25^,^26^, and OS binding to RPE cells evokes cytosolic Ca^2+^ spikes in RPE cells^27^, it is reasonable to hypothesize that molecular clock oscillations and intracellular Ca^2+^ signaling in RPE cells are involved in the generation of intrinsic disc-shedding rhythms. However, substantial evidence is lacking to prove this process.

Our group focused on interactions between clock gene transcriptional rhythms and cellular physiological rhythms using long-term Ca^2+^ measurements with yellow cameleon (YC) Ca^2+^ sensor proteins^28^,^29^,^30^,^31^. Here, to address molecular and cellular activity rhythms in the RPE, we established a human RPE cell line (hRPE-YC) that stably co-expressed *Bmal1-luciferase*^19^ and the *YC3.6* Ca^2+^ sensor^30^. Using hRPE-YC cells, we visualized interactive rhythms in *Bmal1* transcriptions, cytosolic Ca^2+^, and phagocytic activities in these cells. In addition, because we observed consistent cytosolic Ca^2+^ mobilizations via *M3* muscarinic acetylcholine receptors in hRPE-YC cells, the effect of a muscarinic agonist (carbamylcholine, carbachol) on phase responsiveness in *Bmal1-luciferase* rhythms was analyzed in detail.

## Results

### Functional expression of *M3* muscarinic acetylcholine receptors

**Figure 1:**
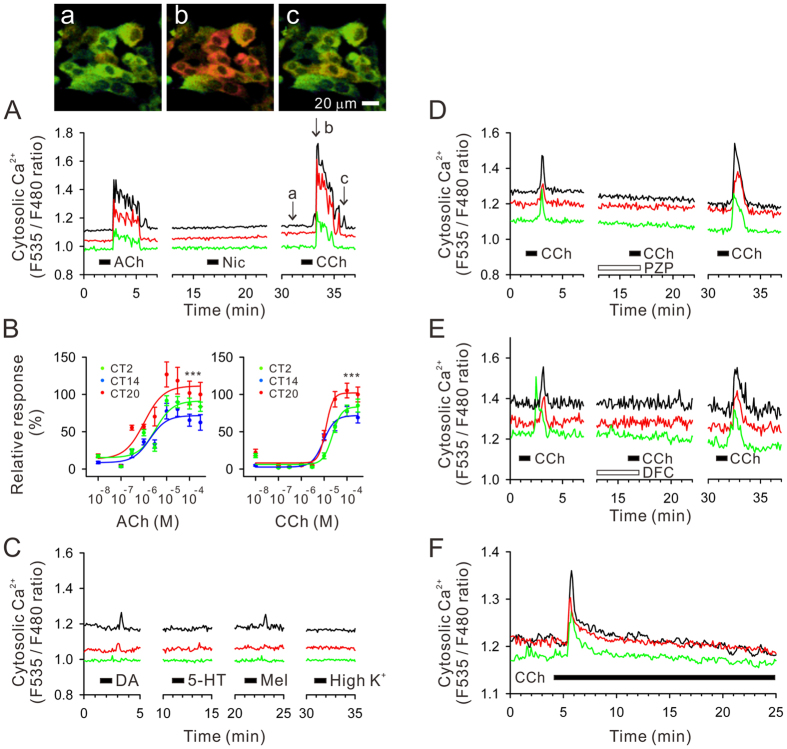
Cytosolic calcium mobilizations in hRPE-YC cells. (**A**) Acetylcholine (ACh, 100 μM) and the muscarinic receptor agonist carbachol (CCh, 100 μM), but not nicotine (Nic, 100 μM), evokes Ca^2+^ transients in hRPE-YC cells. Three representative cell responses are shown. Black bars under the traces denote the timing of perfusion bulb switching, prior to actual drug delivery for 1 min. Virtual color Ca^2+^-concentration images at three timings specified in CCh session (a–c) are shown on the top. (**B**) The concentration-response curves for ACh and CCh were analyzed at three different CTs. The magnitudes of Ca^2+^ responses were largest at CT20 both for ACh and CCh stimulations. ***P < 0.001 by two-way ANOVA. (**C**) Apparent Ca^2+^ mobilizations were not produced by 100 μM dopamine (DA), 100 μM serotonin (5-HT), 100 μM melatonin (Mel), and 80 mM high potassium (High K^+^). (**D**) The CCh-induced Ca^2+^ mobilizations were inhibited by 10 μM pirenzepine (PZP). (**E**) The CCh-induced Ca^2+^ mobilizations were also inhibited by 10 μM darifenacin (DFC). (**F**) The CCh (50 μM)-induced Ca^2+^ mobilizations depended on the onset of CCh stimulations, but were not enhanced by continuous stimulations. All above experiments were reproducible for at least three independent trials in separate culture dishes.

Live hRPE-YC cells were stimulated with various receptor agonists and the responses were screened by Ca^2+^ imaging. Of these, cholinergic reagents, acetylcholine and carbachol, increased cytosolic Ca^2+^ in nearly all hRPE-YC cells examined (number of cells = 272 in seven separate experiments; Fig. 1A). The acetylcholine and carbachol-induced Ca^2+^ elevations were both concentration-dependent with EC_50_ values of 1.0–3.3 μM and 9.4–22.9 μM, respectively (Fig. 1B). The magnitude of the Ca^2+^ responses was also analyzed as a function of circadian time (CT), which is defined by the average *Bmal1-luciferase* rhythms in a culture dish. The magnitude of the Ca^2+^ response was greater at CT20 (the time point with peak chemiluminescence in the *Bmal1-luciferase* rhythms) than at CT2 or CT14 (F_2,89_ = 23.61; *P* < 0.001 by two-way ANOVA), whereas the EC_50_ values were not significantly changed across the circadian cycle (Fig. 1B). Conversely, nicotine (100 μM), dopamine (100 μM), serotonin (100 μM), melatonin (100 μM), and high potassium (80 mM) failed to produce apparent Ca^2+^ mobilizations (number of cells = 286 in seven experiments; Fig. 1A,C). The carbachol-induced Ca^2+^ mobilization was completely abolished by the general muscarinic antagonist pirenzepine (10 μM; number of cells = 115 in three experiments; Fig. 1D) or by the *M3* selective antagonist darifenacin (10 μM; number of cells = 119 in three experiments; Fig. 1E). The decay time constant in carbachol-induced Ca^2+^ mobilization was not modified by continuous (20 min) exposure to carbachol (number of cells = 124 in three experiments; Fig. 1F), indicating rapid desensitization of receptors following carbachol stimulations.

**Figure 2:**
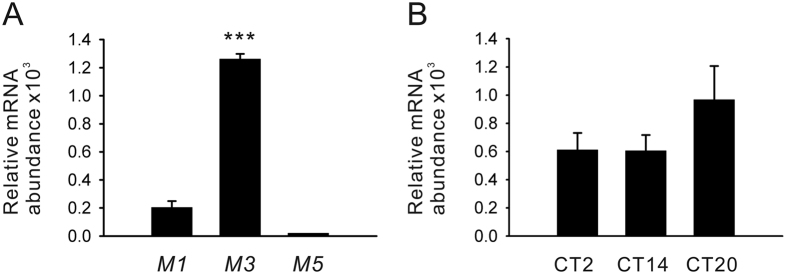
Transcriptional profiles of muscarinic receptor subtypes. (**A**) Within G_q_-coupled muscarinic receptor subtypes, *M3* is the dominant subtype in hRPE-YC cells. Relative mRNA abundance was evaluated by real-time RT-PCR. ***P < 0.001 by one-way ANOVA. A housekeeping gene (*β-actin*) was evaluated to estimate relative expression levels. Cells were sampled during subjective nighttime (CT12–24). (**B**) The gene expression levels of *M3* receptors at three different CTs. Although the levels tend to be greater at CT20, this difference was not statistically significant.

Gene expression profiles of G_q_-coupled muscarinic receptors (*M1, M3*, and *M5*) were also analyzed in hRPE-YC cells using real-time RT-PCR. The highest gene expression was observed for the *M3* subtypes (F_2,21_ = 232.5; *P* < 0.001 by Duncan’s multiple range test following one-way ANOVA; Fig. 2A). The expression of *M3* receptor genes tended to be greater at CT20, whereas the difference was not statistically significant (F_2,18_ = 0.04; n.s. by one-way ANOVA; Fig. 2B).

### Circadian rhythms in spontaneous Ca^2+^ spiking frequencies

**Figure 3:**
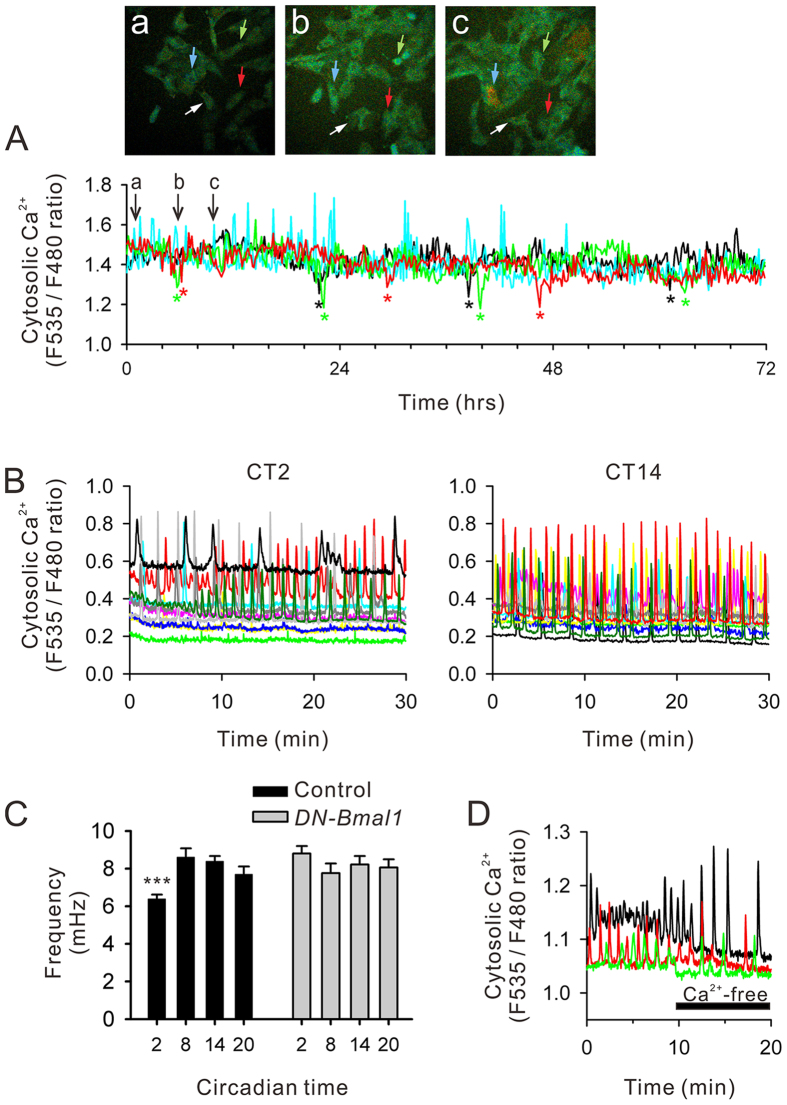
Long-term Ca^2+^ imaging. (**A**) Conventional 3-day imaging of cytosolic Ca^2+^ demonstrated an absence of circadian rhythms in baseline cytosolic Ca^2+^ levels in hRPE-YC cells. Red, green, blue, and black traces correspond to continuous cytosolic Ca^2+^ changes in four cells pointed by red, green, blue, and white arrows in virtual color images on the top (a–c). Interestingly, temporal reduction in cytosolic Ca^2+^ levels immediately before and after cell divisions was observed at approximately 24-h cycles (asterisks). Additionally, intrinsic Ca^2+^ spiking activities were occasionally monitored, whereas it was hard to visualize their circadian rhythmicity by this analysis. (**B**) Ca^2+^ spiking frequencies were further analyzed for 30 min at CT2 or CT14 using a two-photon confocal microscope at higher sampling rate. Each colored trace represents the Ca^2+^ spiking profile from single cell. (**C**) Analysis at four different CTs demonstrated the lowest Ca^2+^ spiking frequency at CT2. ***P < 0.001 by one-way ANOVA. Transfection of *DN-Bmal1* canceled circadian variations in Ca^2+^ spiking frequency. (**D**) Temporal replacement of extracellular buffer to Ca^2+^-free buffer failed to inhibit Ca^2+^ spikes, indicating store-driven Ca^2+^ spikes in these cells.

Physiological activity rhythms in hRPE-YC cells were estimated by long-term Ca^2+^ imaging techniques. The continuous fluorescent ratio monitoring at a low sampling rate (image pair acquisition per 10 min) demonstrated that there were no circadian rhythms at baseline Ca^2+^ concentrations (number of cells = 53 in three experiments; Fig. 3A, Supplementary Movie 1). Interestingly, a transient decrease in cytosolic Ca^2+^ concentration was observed immediately before and after cell divisions (Fig. 3A; Supplementary Movie 1). In addition, intrinsic Ca^2+^ spiking activities were observed in hRPE-YC cells, whereas the sampling rate in long-term Ca^2+^ imaging could not precisely report the amplitude and frequency of Ca^2+^ spikes. Therefore, we further analyzed the population average of Ca^2+^ spiking frequencies at a higher sampling rate with minimized photo-toxicity using two-photon microscopy (Fig. 3B). The results demonstrated that the Ca^2+^ spiking frequencies in hRPE-YC cells were smaller during early subjective day (CT2) than during other circadian time points (F_3,893_ = 12.98; *P* < 0.001 by Duncan’s multiple range test following one-way ANOVA; Fig. 3C). The circadian variations in the Ca^2+^ spiking frequencies were abolished (Fig. 3C; n.s. by one-way ANOVA) by transfection of dominant-negative *Bmal1 (DN-Bmal1*), which reduced the amplitude of *Bmal1* transcription rhythms by nearly 70% (Supplementary Fig. 1). The spontaneous Ca^2+^ spikes in hRPE-YC cells were caused by the release of Ca^2+^ from internal Ca^2+^ stores, because switching the regular extracellular buffer to Ca^2+^-free buffer did not block Ca^2+^ spikes for 10 min (number of cells = 42 in three experiments; Fig. 3D). Thus, these results indicate the presence of Ca^2+^ spiking rhythms driven by clock gene transcriptions and Ca^2+^ release from internal Ca^2+^ stores.

### Carbachol and forskolin, but not light-pulse, phase-shift the *Bmal1-luciferase* rhythm

**Figure 4:**
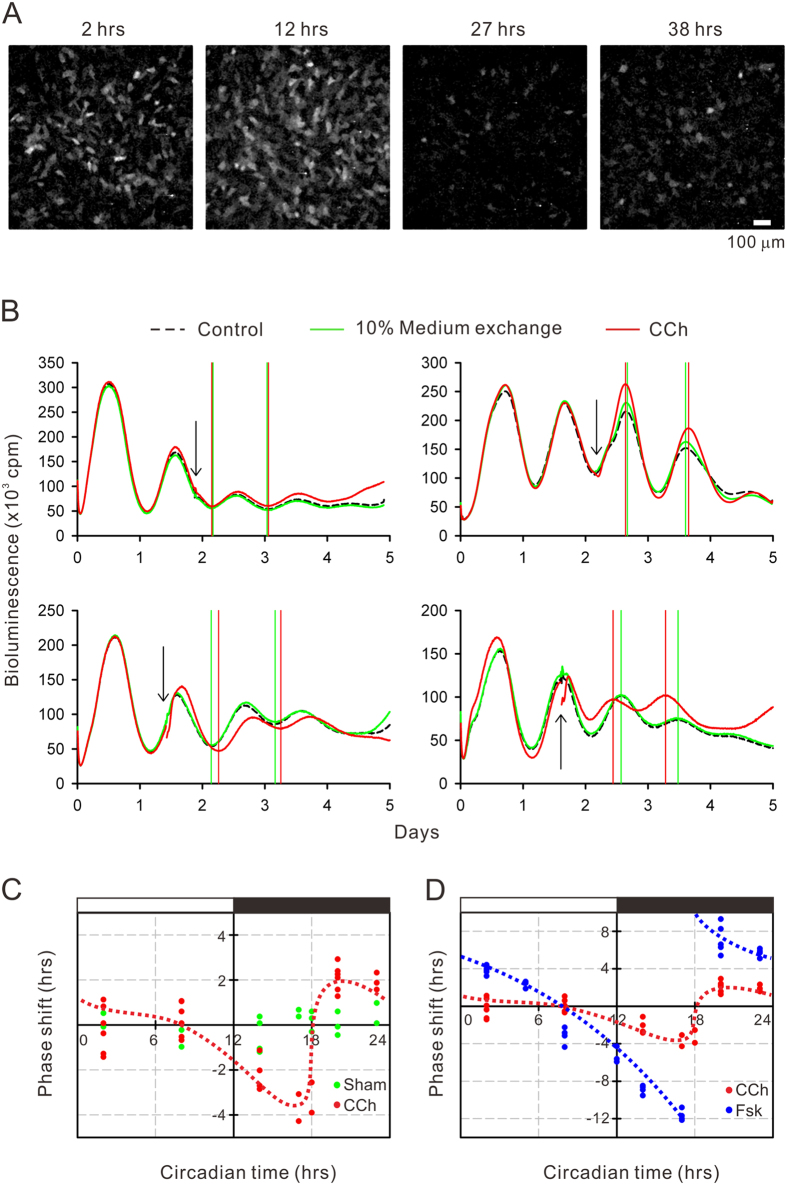
Bmal1 transcriptional rhythms. (**A**) The *Bmal1-luciferase* chemiluminescent images of hRPE-YC cells at four different time points after a 1 μM dexamethasone pre-treatment. Note that synchronous and cyclic intensity changes were observed. (**B**) The *Bmal1-luciferase* intensity (average in 35-mm dish) was quantified using a multi-channel chemiluminescent analyzer. Arrows indicate onset of 50 μM carbachol (CCh) exposures. Subsequent troughs or peaks of circadian waves were compared with groups with non-treated controls. (**C**) Based on the CCh-induced phase shifts at various time points, a type-1 PRC was successfully fitted (red dotted line). The sham-treated group (10% v/v medium exchange) failed to induce significant phase shifts (green circles). (**D**) The identical analysis, but with 5 μM forskolin (Fsk), produced larger phase shifts and formed the type-0 PRC (blue dotted lines).

To analyze molecular clock behaviors, *Bmal1-luciferase* activities were visualized using a chemiluminescent imager. Following 1 μM dexamethasone treatment, hRPE-YC cells displayed synchronous induction of chemiluminescence that oscillated with circadian period (Fig. 4A). Thus, to analyze the field intensity changes during circadian cycles, we used an eight-channel luminometer for the following analyses. On the second day after monitoring, carbachol was applied to dishes by collection of 10% (v/v) of the culture medium, which was returned to the dishes with carbachol (the final diluted concentration was 50 μM). Compared with the *Bmal1-luciferase* rhythms without carbachol supplementation, dishes with carbachol displayed phase-shifted rhythms in the subsequent circadian cycle depending on the timing of applications; application at CT14 produced phase delays and application at CT20 produced phase advances (Fig. 4B). By quantifying phase gaps as a function of timing of carbachol applications, a phase response curve (PRC) was fitted (Fig. 4C). The fitting curve obeyed a typical type-1 PRC for carbachol stimulations (Fig. 4C).

**Figure 5:**
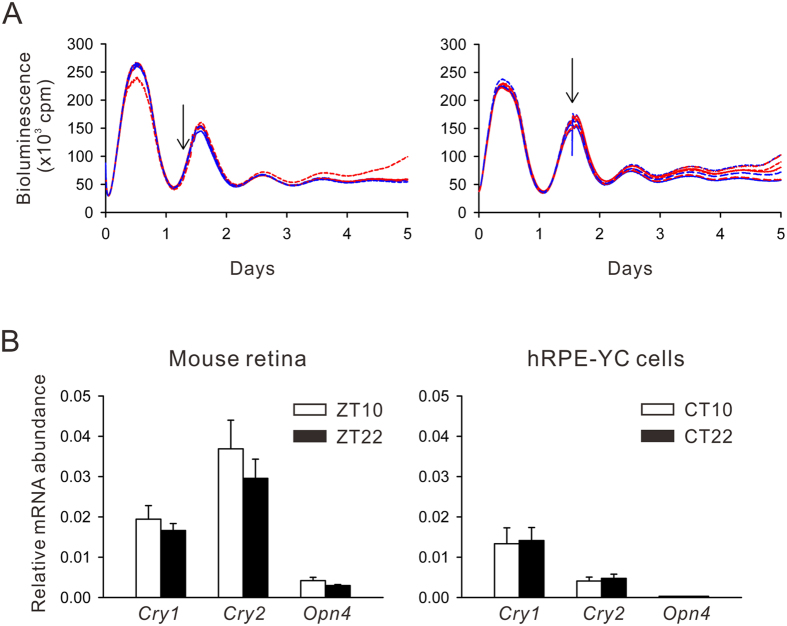
Lack of light pulse-induced phase shifts in hRPE-YC cells. (**A**) *Bmal1-luciferase* recordings as in Fig. 4B, but with a 5 min light pulse (2,000 lux, arrows). The light pulse exposures failed to induce phase shifts (red traces) compared with cells without light exposures (blue traces). (**B**) Gene expression profiles of *Cry1, Cry2*, and *Opn4* were analyzed using real-time RT-PCR in total mouse retina (*left*) and hRPE-YC cells (*right*). These gene expression levels did not depend on the time of day of tissue sampling (ZT10, 2 h before dark onset; ZT22, 2 h before light onset) or CTs in hRPE-YC cell cultures. Note that *Cry2* levels were significantly smaller and *Opn4* levels were negligible in hRPE-YC cells.

Using the same experimental paradigm, effects of forskolin (5 μM) were also analyzed. Forskolin shifted *Bmal1-luciferase* rhythms to the same direction of carbachol stimulation with larger magnitudes. Eventually, the type-0 PRC was fitted to the forskolin-induced phase shifts (Fig. 4D). A 10% v/v culture medium exchange occasionally produced small circadian phase shifts, with no apparent direction. The non-specific phase shifts were not due to temporal dim lighting (<5 lux) during medium exchanges, because bright light exposure (2,000 lux for 5 min) at corresponding timing failed to produce apparent phase shifts (Fig. 5A). Little photosensitivity in hRPE-YC cells may be due to limited expression of photo-sensor molecules, such as *Cryptochrome* 2 (*Cry2*) and *melanopsin (Opn4*), in these cells (Fig. 5B).

**Figure 6:**
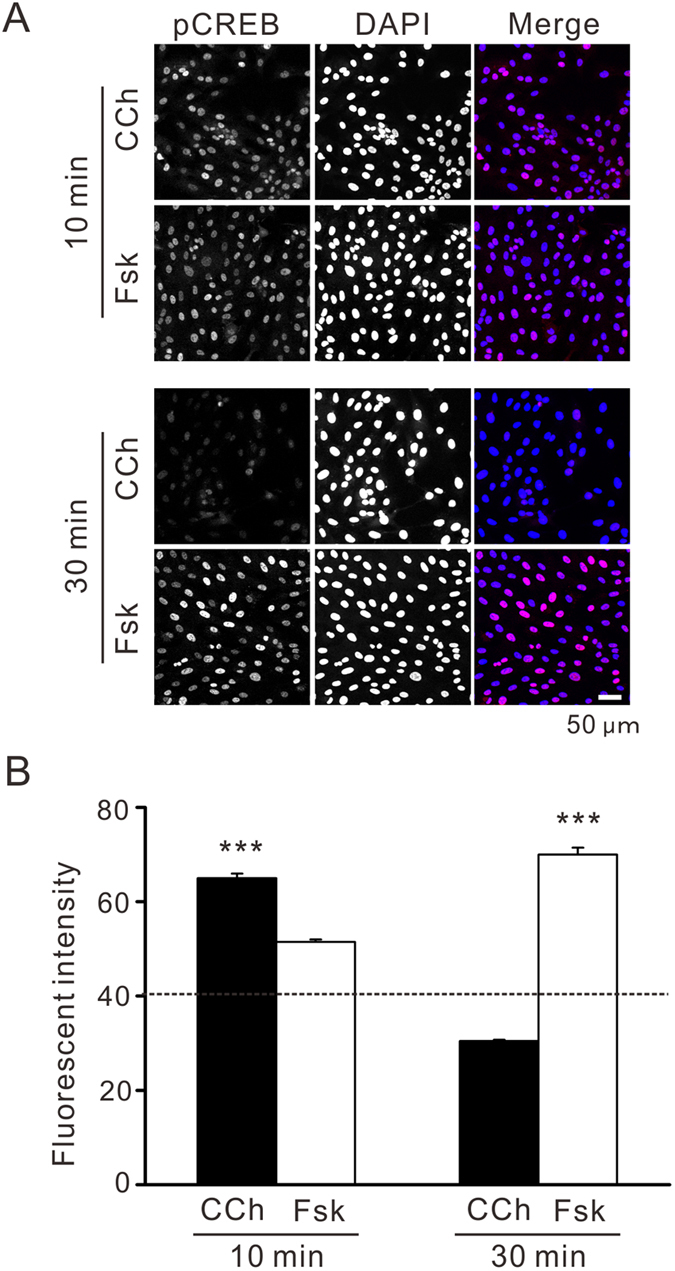
Differential CREB phosphorylation levels following carbachol (CCh) and forskolin (Fsk) exposure. (**A**) Immunofluorescent staining of pCREB (red color in merged picture) following 10 or 30 min exposure to CCh or Fsk. Counter-staining using DAPI (blue color in merged picture) demonstrates the nuclear localization of pCREB signals in hRPE-YC cells. (**B**) Nuclear pCREB levels were quantified as a function of nuclear fluorescent intensity. Dashed line denotes the average intensity of unstimulated cells. Each average was calculated from 300–500 cells in at least three separate experiments. Significant differences were found between the CCh and Fsk groups. ***P < 0.001 by two-tailed Student’s *t*-test.

To address the mechanisms underlying the differential PRCs induced by carbachol and forskolin, the phosphorylation levels of cAMP response element-binding protein (CREB) were analyzed by immunocytochemistry in hRPE-YC cells (Fig. 6A). Immediate (<10 min) nuclear elevation in phosphorylated CREB (pCREB) was observed after both the carbachol and forskolin applications, with a slightly larger elevation after carbachol stimulation (+26% fluorescent intensity; *P < *0.001 by two-tailed Student’s *t*-test; Fig. 6B). However, the carbachol-induced nuclear pCREB elevation was transient and the level was even lower (−24.5% fluorescent intensity) than that of unstimulated hRPE-YC cells following a 30-min exposure to carbachol (*P < *0.001 by two-tailed Student’s *t*-test; Fig. 6B). Conversely, the forskolin-induced nuclear pCREB elevation was enhanced following 30 min of exposure, and the level was 2.3-fold higher than that after carbachol exposure (*P < *0.001 by two-tailed Student’s *t*-test; Fig. 6B). Thus, these results indicate that the duration of the nuclear pCREB induction, which depends on the stimulant, determines the type of PRCs in hRPE-YC cells.

### Regulation of phagocytic activities by circadian clock and carbachol

**Figure 7:**
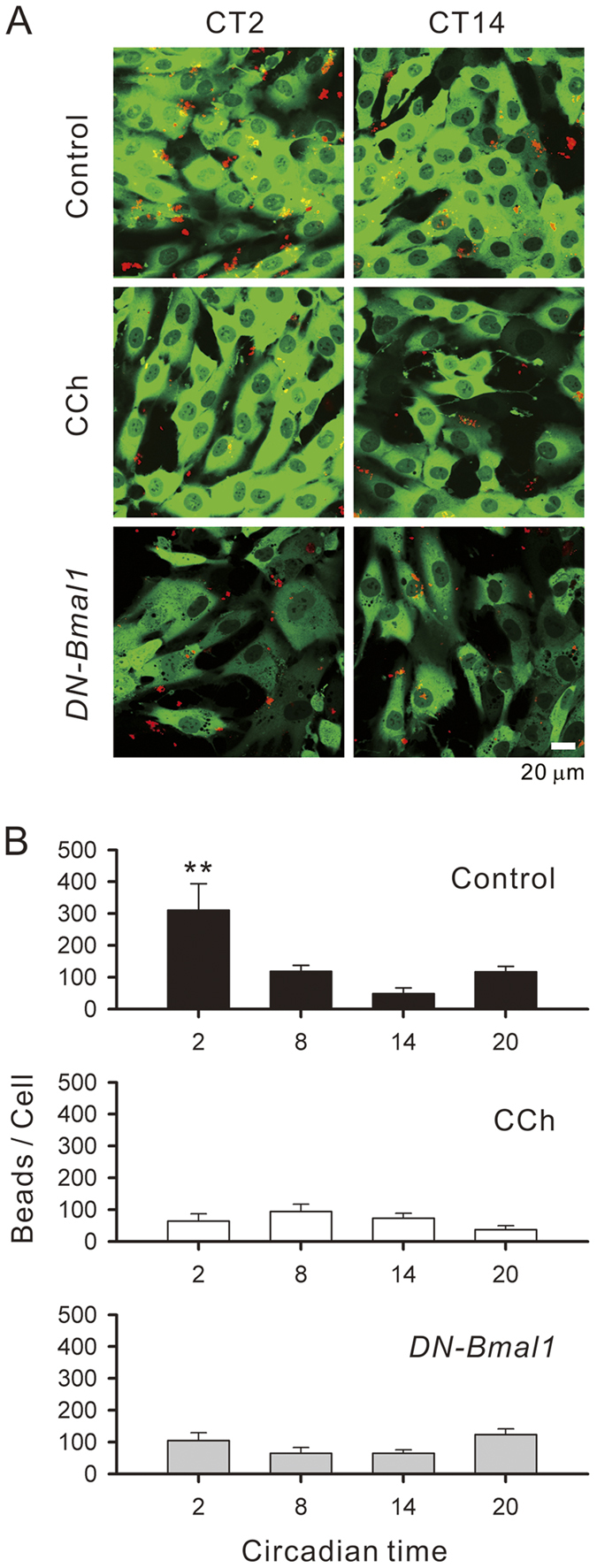
Phagocytic activity rhythms in hRPE-YC cells. (**A**) *Upper.* Three-hour phagocytic activities of the fluorescent latex beads (red spots) were compared between early subjective day (CT2) and early subjective night (CT14). Green fluorescence of hRPE-YC cells was used to estimate the field cell density. Because phagocytosis generally occurred at fine cellular projections, red spots were visualized outside of green fluorescence. *Middle.* Similar analysis as above, but with 50 μM carbachol (CCh) pre-treatment. *Lower*. Similar analysis of controls, but with cells transfected with *DN-Bmal1*. (**B**) The relative intensity of red fluorescence was analyzed as an index of phagocytic activity at four different CTs. The largest phagocytic activity was found at CT2. Circadian variations in phagocytic activities were abolished either by CCh pretreatment or *DN-Bmal1* transfection. Means and standard errors were calculated from 4 independent trials for each group. **P < 0.01 by one-way ANOVA.

Phagocytic activities of hRPE-YC cells were analyzed by culturing cells with red fluorescent latex beads for 3 h, followed by removal of extracellular fluorescent signals by rinsing and quenching immediately before optical quantification. With confocal microscopy, red spots were visualized at the tip of cellular projections (Fig. 7A). The number of red fluorescent beads that internalized from late subjective night (CT23) to early subjective day (CT2) was significantly larger than that at other CTs (analysis based on seven to eight imaging fields in three dishes; F_3,26_ = 5.96, *P* < 0.01 by Duncan’s multiple range test following one-way ANOVA; Fig. 7A,B). Interestingly, when cells were stimulated with 50 μM carbachol 30 min prior to image acquisition, the large fluorescent signal at CT2 significantly reduced to the level observed at other CTs, resulting in loss of circadian variations (n.s. by one-way ANOVA). In addition, a similar analysis in hRPE-YC cells that underwent transfection of *DN-Bmal1* failed to display apparent circadian variations in phagocytic activity levels (n.s. by one-way ANOVA).

## Discussion

In the earlier work for a human RPE cell line^19^, *Bmal1* transcriptional rhythms, whose periods were lengthened by high concentrations of lithium, were characterized, whereas the mechanisms by which temporal cues directly regulate transcriptional and cellular activity rhythms were not described. By generating hRPE-YC cells, we were able to visualize interactive rhythms in *Bmal1* transcriptions, cytosolic Ca^2+^, and phagocytic activities in these cells. It has been described that phagocytosis of RPE cells determines disc shedding of photoreceptor OS. Therefore, we suggest that molecular clock oscillations and cytosolic Ca^2+^ rhythms in RPE cells may be involved in disc-shedding rhythms. In addition, the present results suggest that the cholinergic system in the eyes is involved in the regulation of the circadian rhythm in RPE cells.

Daily photoreceptor disc shedding underlies circadian rhythms in photic sensitivities, yet the mechanism remains unclear. Phagocytosis of photoreceptor OS in RPE cells is triggered by light. Large phagosomes have been observed at the light-onset time in daily light-dark cycles^6^,^32^,^33^. In addition, circadian rhythms of disc shedding and phagocytosis persist in constant darkness^6^,^7^,^8^, suggesting the presence of intrinsic oscillatory mechanisms for the control of disc-shedding rhythms. Photic signal to control phagocytic rhythms could be processed within the eye and not via central circadian clock outputs because a SCN lesion failed to modulate phagocytic rhythms in RPE cells^7^. As discussed in a recent review paper by McMahon^5^, it is still poorly understood whether the disc-shedding rhythms are produced by retinal photoreceptor cells or with interaction to RPE cells. The present results demonstrated (i) the presence of *Bmal1*-dependent rhythms in Ca^2+^ spiking frequencies and phagocytic activities and (ii) the absence of photic regulation in *Bmal1* transcriptional rhythms in hRPE-YC cells. Although *DN-Bmal1* may affect not only the clockwork, but also diverse intracellular events including metabolic controls^34^, and the present data were derived from a single cell line, it is reasonable to hypothesize that (i) intrinsic Ca^2+^-spiking rhythms in RPE cells may be involved in disc-shedding rhythms and (ii) photic regulation of phagocytic activities in RPE cells may be conducted via photoreception outside the RPE cells and subsequent signal transduction from photoreceptors to RPE cells. Although latex bead phagocytosis does not depend on a specific binding process to photoreceptor OS^35^, the intrinsic rhythms in the RPE cells suggested in this study could be a strong driving force for circadian disc-shedding rhythms.

In addition to the intrinsic (i.e., *Bmal1*-dependent) phagocytic activity rhythms, the present results demonstrated carbachol-induced inhibition of phagocytic activities in hRPE-YC cells. It has been recently shown that gene knockout of L-type Ca^2+^ channels (Ca_v_1.3−/−) partially decreased light-onset phagocytic activities, but increased midday phagocytic activities in *in vivo* RPE cells^36^. Notably, carbachol action found in the present study was far beyond the effect of Ca_v_1.3−/−, which resulted in the cancellation of intrinsic phagocytic rhythms. The presence of choline acetyltransferase and high-affinity choline transporter in photoreceptor OS has been shown^37^, and thus such inter-retinal networks may underlie the regulatory pathway. *M3* muscarinic receptor couples phospholipase C (PLC) to mobilize intracellular Ca^2+^ 38,39. Importantly, it has shown that photoreceptor disc shedding and the phagocytic process in RPE cells are also associated with phosphoinositide signaling^40^: the process begins with shedding of photoreceptor OS discs, which expose phosphotidylserine at the tips of photoreceptors^41^ to facilitate binding to αvβ5 integrin^42^,^43^ and CD36^44^ on the apical surface of RPE cells. Subsequently, the Mer tyrosine kinase is used for internalization^45^, and this process may coincide with PLC activation, which promotes inositol triphosphate/diacylglycerol production and intracellular Ca^2+^ mobilization^40^. Indeed, metabotropic Ca^2+^ spiking follows OS binding to RPE cells^27^. Involvement of Ca^2+^ release from internal stores was also suggested as a knockdown of bestrophin-1, a Ca^2+^/Cl^−^ co-transporter at the endoplasmic reticulum, partially increased phagocytic activities of RPE cells^36^,^46^. Because the present results demonstrated carbachol-induced inhibition of phagocytic activities, large cytosolic Ca^2+^ mobilizations may be involved in the termination of phagocytic processes. This interpretation is consistent with dual roles of Ca^2+^ for the regulation of phagocytic activities in RPE cells, which were hypothesized using Ca_v_1.3−/− mice^36^.

The present results also demonstrated muscarinic regulation of *Bmal1* transcriptional rhythms in hRPE-YC cells. Magnitudes of Ca^2+^ mobilizations via acetylcholine and carbachol were not significantly different between subjective day and night. Thus, the refractory period in the PRC during the subjective day is apparently not a matter of the size of Ca^2+^ mobilizations, but may be more downstream events linking to the gene transcriptions. It has been shown that *Per2-luciferase* rhythms were phase-shifted in type-1 and type-0 PRCs, depending on the duration of light exposure (0.5–12 h) to NIH3T3 mouse fibroblasts overexpressing G_q_-coupled photo-sensor melanopsin^47^. Although how light exposure impacts net cytosolic Ca^2+^ levels was not described in that study, it has been suggested that the duration of cytosolic Ca^2+^ mobilizations could be the determinant for types of PRCs. In the present study, carbachol and forskolin were continuously bath-applied to cells after specific time points, whereas carbachol-induced phase-shifting profiles were similar to PRCs via short-term light exposures in the NIH3T3 fibroblast models^47^. This may be due to rapid desensitization of muscarinic receptors as continuous carbachol stimulation, which evoked only the onset Ca^2+^ rise in hRPE-YC cells.

Forskolin, which is lipophilic and a direct activator for adenylyl cyclase, produced further larger phase shifts in the same experimental paradigm and formed type-0 PRC, similar to the results with longer light exposures to melanopsin-expressing fibroblasts^47^. Exposure of melanopsin-expressing fibroblasts to light induces phosphorylation of CREB^48^, which is known to take place in the SCN of hamsters following nocturnal light exposure^49^. Taken together, it is reasonable to assume that intracellular signaling underlying phase responses in hRPE-YC cells may also take place via phosphorylation of CREB, which is induced either by forskolin (i.e., via cAMP-dependent protein kinase) or by carbachol (i.e., via Ca^2+^/calmodulin-dependent protein kinase). Indeed, we observed (i) a transient Ca^2+^ elevation and pCREB induction upon carbachol exposure, and (ii) a long-lasting pCREB induction following forskolin exposure. The difference in the PRC shape upon carbachol and forskolin stimulation can thus be explained by the duration and magnitude of CREB phosphorylation. It has been shown that the human circadian clock is phase shifted by single or repeated light exposure and formed type-1 or type-0 PRCs, depending on the intensity of stimulus or light exposure paradigm^50^,^51^,^52^. Thus, the present results suggest that such human clockwork could be modeled by intracellular signaling levels using hRPE-YC cells.

Day-night and/or circadian rhythms in photoreceptor OS phagocytosis have been extensively explored in many species. Compared with cone phagocytosis, rod phagocytosis more regularly occurs shortly after light onset, regardless of diurnal and nocturnal species, as in mice^53^, rats^6^, ground squirrels^54^, and rhesus monkeys^55^. Thus, it is possible that intrinsic phagocytic rhythms in hRPE-YC cells are involved in general rod disc-shedding rhythms. Mutant mice lacking functional rods, but retaining both cone and melanopsin phototransduction pathways, exhibit impaired photoentrainment at a wide range of light levels^56^. Thus, rods play crucial roles for circadian photoentrainment. Maintenance of rod functions by RPE cells, therefore, could be important for photic input pathways to the central circadian clock, as well as clock works within the eye. The generation of a conditional knockout of *Bmal1* in RPE cells may provide further insights into the total contribution of RPE clocks for the systems circadian clock, although the study is beyond the scope of current study.

The mechanisms involved in disturbance of the human biological clock and resultant diseases, such as circadian rhythm sleep disorders, remains poorly understood. Thus, the human circadian clock system has been extensively analyzed from clinical aspects and by physiological studies on healthy volunteers, yet studies on the cellular and molecular machineries remain limited. For example, the transcriptional rhythm of the clock gene *Per3* in leukocytes may be coupled to the donor’s (system’s) clock movements, because they are phase advanced by exposure of the donor to light at appropriate timing^57^. In addition, recent studies have demonstrated a particular correlation between clock gene transcriptional rhythms in human fibroblasts and sleep-wake profiles in fibroblast donors^58^,^59^,^60^. However, fundamental information is lacking on human cellular clockwork, because substantial regulatory mechanisms producing phase responses have not yet been demonstrated in cell culture models. In this regard, the present results provide a powerful model for understanding the intracellular signaling mechanisms underlying or regulating human cellular clockwork.

In conclusion, the present results provide a cellular model for understanding the molecular and intracellular signaling mechanisms underlying the human retinal circadian clock and propose a *de novo* function of the cholinergic system in human eyes.

## Methods

### Generation of hRPE-YC clones

Human immortalized RPE cells stably expressing *Bmal1-luciferase* (hRPE cells) were generated from the hTERT-immortalized human retinal pigment epithelial cell line (hTERT RPE-1; purchased from the American Type Culture Collection, Manassas, VA)^19^. The hRPE cells were cultured with Dulbecco’s-Modified Eagle Medium/F12 (DMEM/F12) supplemented with 10% FBS (Invitrogen, Carlsbad, CA), sodium bicarbonate (1.2 g/L), and 1% penicillin/streptomycin antibiotics (Invitrogen) under constant temperature (37 °C) and 5% CO_2_.

Because hRPE cells^19^ are resistant to neomycin, the *YC3.6* gene was ligated to the multiple cloning site of a zeocin-resistant vector (pcDNA3.1/zeo; Invitrogen) and transfected into hRPE cells using Lipofectamine-2000 (Invitrogen). Subsequently, the cells were cultured in medium containing zeocin (400–800 μg/mL) for cell selection. Four colonies grown from single cells were picked up by a cloning ring for further subcloning using 96-well plates. Finally, one clone steadily expressing *YC3.6* was used in the present study.

### Ca^2+^ imaging

For the receptor screening assay, the cells were seeded onto 35-mm glass-bottom dishes and cultured as described above in a CO_2_ incubator (cell density: 2–4 × 10^5^ cells/dish). The culture medium was gently rinsed from the dishes using buffered salt solution (BSS) consisting of (in mM) 128 NaCl, 5 KCl, 2.7 CaCl_2_, 1.2 MgCl_2_, 1 Na_2_HPO_4_, 10 glucose, and 10 HEPES/NaOH (pH 7.3). hRPE-YC cells were placed on an inverted microscope stage (TE300; Nikon, Tokyo, Japan) and continuously perfused with BSS at a flow rate of 2 mL/min through an in-line heater (SF-28; Warner Instruments, Hamden, CT) set at 36 °C. Fluorescent image pairs (535 ± 15 nm and 480 ± 15 nm) were produced by a 440 ± 5 nm light pulse (300 msec pulses generated by Lambda-LS 300 W Xenon lamp house, Sutter Instrument, Novato, CA), which was conducted to the microscope through a liquid light guide and reflected using a dichroic mirror (FT 445 nm). These images were acquired using a cooled CCD camera (CoolSnap Fx, Photometrics, Tucson, AZ) through a 20× objective lens (Plan Fluor 20×/0.50, Nikon) and a filter wheel (Lambda 10-3, Sutter Instrument) attached in front of the camera. Timings of shutter gating and image acquisitions at 6-s intervals were regulated by digital imaging software (MetaFluor ver. 6.0; Japan Molecular Devices, Tokyo, Japan). The background fluorescence was also subtracted using the software. Dopamine hydrochloride (Sigma-Aldrich, St. Louis, MO), serotonin hydrochloride (Sigma), melatonin (Sigma), L-glutamate monohydrate (Wako Pure Chemical Industries, Ltd., Osaka, Japan), acetylcholine chloride (Sigma), carbamylcholine chloride (Sigma), nicotine hemisulfate salt (Sigma), pirenzepine dihydrochloride (Abcam, Cambridge, UK), and darifenacin hydrobromide (LKT Laboratories, Inc., St. Paul, MN) were delivered to the cells by switching the perfusate. For analysis of CT dependencies in cholinergic Ca^2+^ responses, *Bmal1-luciferase* rhythms were monitored as below by the luminometer system. Following online estimation of chemiluminescent rhythms, culture dishes were replaced to the Ca^2+^ imaging system and stimulated with acetylcholine or carbachol at different concentrations to obtain concentration response curves.

The following two approaches were used to analyze circadian rhythms in cytosolic Ca^2+^ concentrations. First, hRPE-YC cells plated as above on glass-bottom dishes were treated for 1 h with 1 μM dexamethasone (Sigma) before recordings. Subsequently, cells were rinsed and cultured with fresh culture medium. The culture dish was kept in a temperature- and CO_2_-controled (36 ± 0.5 °C and 5 ± 0.2%) custom-built chamber, which enclosed an entire inverted microscope (DM-IRB; Leica Microsystems, Tokyo, Japan). An optical fiber light source (EXFO Photonic Solutions Inc., Ontario, Canada) set outside the recording chamber was used to supply excitation light. Intensity of excitation light was reduced by 70% using a neutral density filter. The fluorescent images were viewed using a 20× objective lens (HC PL-Fluotar 20×/0.50, Leica) and the same optical filter sets for the receptor screening assay. Fluorescent image pairs were acquired continuously for 3 days at 10-min intervals using a cooled CCD camera (Cascade 1k; Photometrics) and digital imaging software (Image Pro-Plus; Media Cybernetics, Bethesda, MD). The fluorescent intensity was enhanced by 4 × 4 pixel binning and electron-multiplier in the CCD.

Although the above method efficiently analyzed slow baseline Ca^2+^ fluctuations, it was not applicable for the analysis of events faster than the sampling rate. To analyze circadian rhythms in spontaneous Ca^2+^ spike frequencies, therefore, the present study additionally used a multiphoton-confocal laser-scanning microscope (A1MP plus, Nikon). For this analysis, a fluorescent image was acquired by two-photon excitation (880 nm) with a 16× water-immersion objective lens (LWD 16×/0.80, Nikon) and digital imaging software (NIS-Elements AR4.10, Nikon). The fluorescent image pairs (535 ± 15 nm and 480 ± 15 nm) were acquired over 30 min at 2-s intervals separately at four different CTs, normalizing the time after dexamethasone treatment (see below in *Bmal1-luciferase* assay). In this analysis, Ca^2+^ spike frequencies observed in six dishes were compared.

### *Bmal1-luciferase* assay

To monitor the clock gene transcriptional rhythms in hRPE-YC cells, the cells were plated on 35 mm plastic dishes (2 × 10^5^ cells/dish and seven dishes/experiment). On the second day after plating, cells grew at a 90% confluent condition and were treated for 1 h with 1 μM dexamethasone. Subsequently, the culture medium was rinsed with standard culture medium and supplemented with 50 μM beetle luciferin (Promega, Madison, WI). As a blank control, an empty dish filled with the same luciferin-supplemented medium (1 mL) was also prepared. These dishes were light-sealed by aluminum foil and further incubated for 1 h in a CO_2_ incubator prior to recording. Recording chamber of Kronos-Dio luminometer system (Model AB-2550, ATTO Co. Ltd., Tokyo, Japan) was set at 37 °C throughout the experimental period. Duration of photon-counting was set for 1 min per sampling with sampling intervals at 10 min. Background intensity was subtracted using the blank control. The time point with peak chemiluminescent level in the *Bmal1-luciferase* rhythms was regarded as CT20 according to the data based on an *ex vivo* model^61^. To analyze the influence of CTs in the Ca^2+^ imaging and phagocytosis assays, analyses at different CTs were conducted by normalizing the time elapsed after dexamethasone treatment. For example, the data at CT2 were obtained equivalently from two groups at approximately 26 h and 50 h after dexamethasone treatment. Thereafter, the data at CT2 were compared with the data at CT14, which is located between the two groups (approximately 38 h after dexamethasone treatment). The chemiluminescence imager equipped with a deep-cooled electron-multiplier CCD camera (LV200; Olympus, Tokyo, Japan) was used to monitor spatiotemporal distribution of luminescent signals. For this assay, hRPE-YC cells were cultured with 500 μM beetle luciferin and chemiluminescent signals were exposed to the camera for 10 min at 20-min intervals.

To analyze PRCs against pharmacological stimulations, Kronos recordings were paused for 5 min and all dishes were drawn to the on-site clean bench. Ten percent of culture medium (100 μL) was collected from each dish. Carbachol (500 μM) or forskolin (50 μM; Sigma) was added to the collected culture medium and gently returned to the culture dish (final diluted concentration of 50 μM carbachol and 5 μM forskolin). Dishes were kept under dim red light (<5 lux) during initial dish installations and the above medium exchange processes. As controls, dishes underwent a medium exchange without carbachol supplementation or exposing the dishes to bright light (2,000 lux for 5 min). These pharmacological stimulations were examined at the second circadian cycle of *Bmal1-luciferase* rhythms, because the first cycle was strongly influenced by luciferin uptake. Eventual phase-shifts of *Bmal1-luciferase* rhythms at the third to forth circadian cycle were quantified by referring unstimulated controls to the same Kronos recording chamber.

To analyze the effects of *DN-Bmal1*, hRPE-YC cells on 35-mm glass-bottom dishes were transfected with vectors (pcDNA3.1) carrying *DN-Bmal1*^31^ or blank vectors using Lipofectamine-2000 1day prior to the Kronos recording. General transfection rates were 60–75% using Lipofectamine-2000 in hRPE-YC cells and consistent reductions (~70%) in the amplitude of *Bmal1-luciferase* rhythms were observed on the second day or later after *DN-Bmal1* transfections (Supplementary Fig. 1). Using the residual circadian rhythms in *Bmal1-luciferase*, CTs were determined as above and used for the further assay for Ca^2+^ imaging or for phagocytosis.

### Immunofluorescent confocal imaging

To examine the effects of carbachol and forskolin on the CREB phosphorylation levels, hRPE-YC cells plated on 35-mm glass-bottom dishes were stimulated with carbachol (50 μM) or forskolin (5 μM) for 10 or 30 min during subjective nighttime. Immediately after the stimulations, hRPE-YC cells were fixed in 4% phosphate-buffered paraformaldehyde for 15 min and washed three times with phosphate-buffered saline (PBS) consisting of (mM) 137 NaCl, 2.7 KCl, 1.5 KH_2_PO_4_ and 8.1 Na_2_HPO_4_ (pH 7.4). The fixed samples were then incubated for 2 h at room temperature in 10% donkey serum (Jackson Immuno Research Laboratories, West Grove, PA) dissolved in 0.3% Triton-X (Sigma) PBS. Next, samples were incubated with 1:100 affinity-purified rabbit anti-P-CREB (pSer^133^) (Sigma) dissolved in 5% donkey serum PBS for 24 h at 4 °C. After three 20 min PBS rinses, samples were incubated in 1:400 Cy3-conjugated donkey anti-rabbit IgG (Jackson Immuno Research Laboratories) for 2 h at room temperature. Finally, samples were rinsed with PBS (four 15 min rinses on an orbital shaker) and mounted using Vectorshield (Vector Laboratories, Burlingame, CA) containing 4′,6-diamidino-2-phenylindole (DAPI). Images were acquired using a confocal laser-scanning microscope (FV1000, Olympus) with a laser diode (405 nm), helium neon laser (534 nm) and a 20× objective lens (UPL-SAPO 20×/0.75, Olympus). All staining experiments were repeated at least three times. The nuclear immunofluorescent intensity (8-bit depth) was analyzed using Photoshop CS 6 software (Adobe Systems, San Jose, CA).

### Phagocytosis assay

Phagocytic activities were analyzed using a phagocytosis assay kit (Cayman Chemical, Ann Arbor, MI) according to the manufacturer’s instruction. Cells at CT23, CT5, CT11 and CT17 were treated with latex bead-rabbit IgG-Phycoerythrin conjugate (5 μL/1 mL medium/dish) for 3 h. Following rinsing out the beads with standard culture medium, extracellular fluorescence was quenched by 50 μM trypan blue in standard medium. Images were acquired using a FV1000 confocal laser-scanning microscope with argon (488 nm) and helium neon (534 nm) lasers and 60× oil-immersion objective lens (UPL-SAPO 60×/1.35, Olympus). Cytosolic YFP fluorescence from hRPE-YC cells was used to estimate cell shapes and numbers. To analyze the effect of carbachol stimulations, cells were stimulated with 50 μM carbachol 30 min prior to image acquisition according to methods for the *Bmal1-luciferase* assay.

### Real-time RT-PCR assay

The hRPE-YC cells in 35 mm dishes at 90% confluency were rinsed, suspended in PBS, transferred to 1.5 mL RNase-free tubes, and centrifuged for 5 min at 1,000 rpm at room temperature. The cell pellets were transferred to 350 μL of RLT buffer (RNeasy Kit; Qiagen, Chatsworth, CA) and homogenized using a bio-masher (Funakoshi, Tokyo, Japan) at 2,500 rpm for 30 s. The resultant cell lysates were diluted with an equivalent volume of 70% ethanol and stored at −80 °C until RNA extraction. The present study also analyzed transcriptional levels of *Cry1, Cry2*, and *Opn4* in whole mice retina. For this analysis, three 2-month-old male C57BL/6 J mice maintained on a 12 h light/dark cycle at a constant ambient temperature (23 ± 1 °C) were deeply anesthetized with an intraperitoneal injection of sodium pentobarbital (50 mg/kg, body weight). Bilateral eyes were then removed and directly frozen on dry ice. The crystalline lens and vitreous body were carefully removed in ice-cold PBS by stereoscopic surgery, and the remaining bodies, including the sclera, was homogenized as above using a bio-masher. Total RNA (4 μg/sample) was extracted from tissue homogenates using the RNeasy Kit according to the manufacturer’s instructions. The PCR primers used were described in Supplementary information.

Each primer (100 μM) was used in Rotor-Gene SYBR Green RT-PCR Master Mix (Qiagen) according to standard methods. Finally, the PCR amplification was monitored in a strip tube (25 μL reaction volume) set in the 72-well rotor of a real-time PCR system (Rotor Gene 3000 A; Corbett Research, Sydney, NSW, Australia) with the following temperature steps: reverse transcription at 55 °C for 10 min (Hold 1): initial PCR activation at 95 °C for 5 min (Hold 2); and 60 thermal cycles of 95 °C for 5 s and 60 °C for 10 s. The reactions in four separate tubes were averaged for each sample.

### Statistical analysis

Data are presented as means ± standard error. Two-way ANOVA or one-way ANOVA followed by Duncan’s multiple range test were used for statistical comparisons across multiple means. A two-tailed Student’s *t*-test was used for pairwise comparisons. A 95% confidence level was considered to indicate statistical significance. To estimate the concentration-response curve for acetylcholine and carbachol, culture dishes were stimulated twice by low (≤10^−6^ M) and high concentration (>10^−6^ M) drugs with a 20 min gap. Responses in different dishes were averaged and the concentration-response curve was fitted by a four-parameter Hill function. For the chemiluminescent rhythm assay, background intensity was subtracted and general decaying tendency was de-trended using standard Kronos software (ATTO). The PRCs were eye-fitted by three experienced investigators.

## Additional Information

**How to cite this article:** Ikarashi, R. *et al*. Regulation of molecular clock oscillations and phagocytic activity via muscarinic Ca^2+^ signaling in human retinal pigment epithelial cells. *Sci. Rep.* **7**, 44175; doi: 10.1038/srep44175 (2017).

**Publisher's note:** Springer Nature remains neutral with regard to jurisdictional claims in published maps and institutional affiliations.

## Supplementary Material

Supplementary Information

Supplementary Movie S1
