# Supplementary material for: Regulation of molecular clock oscillations and phagocytic activity via muscarinic Ca2+ signaling in human retinal pigment epithelial cells
Source: Sci Rep. 2017 Mar 9;7:44175. doi: 10.1038/srep44175 (PMC5343479; doi:10.1038/srep44175)
Supplement: Supplementary Information [file srep44175-s1.pdf]

Regulation of molecular clock oscillations and phagocytic activity via muscarinic Ca^{2+} signaling in human retinal pigment epithelial cells

Rina Ikarashi*[1], Honami Akechi*[1], Yuzuki Kanda*[1], Alsawaf Ahmad[2], Kouhei Takeuchi[2], Eri Morioka[1], Takashi Sugiyama[3], Takashi Ebisawa[4], Masaaki Ikeda[5,6] & Masayuki Ikeda[1,2]

[1]*Graduate School of Science and Engineering, University of Toyama, 3190 Gofuku, Toyama city, Toyama 930-8555, Japan*
[2]*Graduate School of Innovative Life Science, University of Toyama, 3190 Gofuku, Toyama city, Toyama 930-8555, Japan*
[3]*Advanced Core Technology Department, Research and Development Division, Olympus Co. Ltd., 2-3 Kuboyama, Hachioji, Tokyo 192-8512, Japan*
[4]*Department of Psychiatry, Tokyo Metropolitan Police Hospital, 4-22-1 Nakano, Nakano-ku, Tokyo 164-8541, Japan*
[5]*Department of Physiology, Saitama Medical University, 38 Morohongo, Moroyama, Iruma-gun, Saitama 350-0495, Japan*
[6]*Molecular Clock Project, Project Research Division, Research Center for Genomic Medicine, Saitama Medical University, 1397-1 Yamane, Hidaka city, Saitama, 350-1241, Japan*

SUPPLEMENTARY INFORMATION

Supplementary methods

The PCR primers

human M1 receptor forward	5′-AGACGCCAGGCAAAGGGGGTGG-3′
human M1 receptor reverse	5′-CACGGGGCTTCTGGCCCTTGCC-3′
human M3 receptor forward	5′-GACAGAAAACTTTGTCCACCCCAC-3′
human M3 receptor reverse	5′-AGAAGTCTTAGCTGTGTCCACGGC-3′
human M5 receptor forward	5′-CTCACCACCTGTAGCAGCTACCC-3′
human M5 receptor reverse	5′-CTCTCTTTCGTTTGGTCATTTGATG-3′
human Cry1 forward	5′-CTCCATGGGCACTGGTCTCAGTG-3′
human Cry1 reverse	5′-TCCCCACCAATTTCAGCTGCAAC-3′
mouse Cry1 forward	5′-CCCAGGCTTTTCAAGGAATGGAACA-3′
mouse Cry1 reverse	5′-TCTCATCATGGTCATCAGACAGAGG-3′
human Cry2 forward	5′-CCAAGAGGGAAGGGCAGGGTAGAG-3′
human Cry2 reverse	5′-AGGATTTGAGGCACTGTTCCGAGG-3′
mouse Cry2 forward	5′-GGGACTCTGTCTATTGGCATCTG-3′
mouse Cry2 reverse	5′-GTCACTCTAGCCCGCTTGGT-3′
human Opn4 forward	5′-GGACCGCTACCTGGTAATCA-3′
human Opn4 reverse	5′-GGAGGAAGAACACGAAGCAG-3′
mouse Opn4 forward	5′-CCAGCTTCACAACCAGTCCT-3′
mouse Opn4 reverse	5′-CAGCCTGATGTGCAGATGTC-3′
human β-actin forward	5′-ATGGCCACGGCTGCTTCCAGC-3′
human β-actin reverse	5′-CATGGTGGTGCCGCCAGACAG-3′
mouse β-actin forward	5′-AGTGTGACGTTGACATCCGTA-3′
mouse β-actin reverse	5′-GCCAGAGCAGTAATCTCCTTCT-3′

Supplementary figure 1. Effects of dominant-negative *Bmal1* (*DN-Bmal1*) transfections on *Bmal1* transcriptional rhythms

The *Bmal1* transcriptional rhythms were analyzed by *Bmal1-luciferase* activities using the Kronos-Dio luminometer system. Significant amplitude reductions were observed following *DN-Bmal1* transfections 1 day prior to the regular recording schedule.

Supplementary movie 1. Time-lapse virtual color Ca^{2+} imaging of hRPE-YC cells

This video demonstrates the entire process of 3-day changes in cytosolic Ca^{2+} concentrations shown in Fig. 3A. Warmer color corresponds to higher Ca^{2+} concentrations.

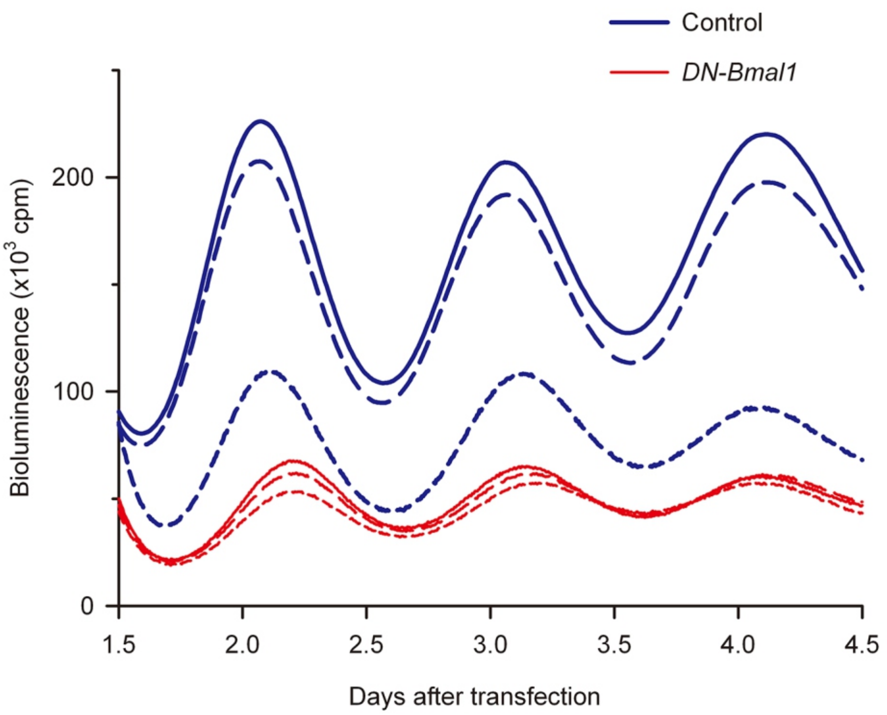

Supplementary Fig. 1
